# Evaluation of the developmental outcome in children with congenital hypothyroidism

**DOI:** 10.22088/cjim.12.3.315

**Published:** 2021-04

**Authors:** Razieh Ehsani, Morteza Alijanpour, Mohammadreza Salehiomran, Farzan Kheirkhah, Leila Moslemi, Faeze Aghajanpour

**Affiliations:** 1 Student Research Committee, Babol University of Medical Sciences, Babol, Iran; 2 Non-Communicable Pediatric Diseases Research Center, Health Research Institute, Babol University of Medical Sciences, Babol, Iran; 3 The Clinical Research Development Unit of Amirkola Children's Hospital, Babol University of Medical Sciences, Babol, Iran; 4 Social Determinants of Health Research Center, Health Research Institute, Babol University of Medical Sciences, Babol, Iran; 5 Health Reproductive Research Center, Sari Branch, Islamic Azad University, Sari, Iran

**Keywords:** Developmental outcome, Congenital hypothyroidism, Pediatrics, Denver Developmental Screening Test II

## Abstract

**Background:**

Congenital hypothyroidism (CH) is one of the most common endocrine diseases and one of the major causes of mental retardation in children. So we aimed to evaluate the developmental outcome of children with CH.

**Methods:**

This case-control study was performed on two 3-6-year-old groups of 100 patients. The case group was children with CH, referred to Endocrine Clinic of Amirkola Children's Hospital (2011-2017) and the control group was healthy children and normal from other states. The Denver developmental screening test-II (DDST_II) was used to assess the developmental factors and disorders in four areas of gross motor, fine motor, personal-social and language. Data were analyzed by SPSS 21 using descriptive statistics, t-test and chi-square, and a p<0.05 was considered significant.

**Results:**

The mean age of 200 children in the case and control groups was 54.62±15.72 and 59.68±15.64 months, respectively. In the case group, 45% and 55% of them had transient and permanent CH, respectively. All four criteria of DDST_II in the control group as well as gross motor in the case group were normal, but fine motor, personal-social and language were reported normal in 94, 95 and 93% of the case group, respectively. All subjects with abnormal DDST_II, had a negative neonatal screening tests.

**Conclusion:**

The results obtained from DDST_II indicated that 6% of children with CH had an abnormal development, all who had an onset of medical treatment over 30 days, which makes it important to screen the neonatal thyroid disease and diagnose this disease timely.

Congenital hypothyroidism (CH) is one of the most prevalent endocrine and metabolic disorders in children (1, 2) that can affect their quality of life (3). If, it is left untreated, it can become a serious threat to a child's health and physical growth and mental development. CH is one of the major causes of mental retardation in children. Before that the heel neonatal thyroid screening becomes prevalent, 1 out of 4000 infants was doomed to suffer the consequences of hypothyroidism. Fortunately, these cases of hypothyroidism in infants who remain untreated are very rare today in the developing countries (4). Thyroid hormone plays an essential role in growth and metabolic homeostasis in humans (5). Thyroid hormone deficiency inhibits full and proper development of brain in children and disrupts physical growth, too (4). According to the studies around the world, the prevalence of hypothyroidism varies. It reaches to 14.7 in Nigeria, to 0.14 in Japan and 0.23 in North America per 1000 infants and with variable prevalence in the provinces of Iran (6-9). For example, the prevalence of hypothyroidism in the north-east center of Iran, was estimated 1/370 (10).

Based on new statistics, the average incidence of hypothyroidism is estimated about 1/3000-4000. This average is 1/1000 in Iran which is higher than the global statistics (11). According to etiology, the hypothyroidism in infants maybe is permanent or transient. After tentative discontinuation of treatment after 3 years of age, if T4 and TSH remain within the normal range, it is regarded as a transient form of CH, otherwise it is considered as a permanent form (8). CH is suspected when TSH is higher than 5miu/l with a heel sample of neonate between 3-7 days of birth, confirmed by Venus sample of TSH higher than 10 miu/l and T4 below than 6.5µg/dl (12).

Hypothyroid neonates have normal appearance at birth and the rate of clinical symptoms is low and nonspecific. Therefore, if the diagnosis is based on these symptoms, diagnosis and treatment are delayed and the infant will be exposed to irreversible complications of the disease as deafness and intellectual retardation (13). 

To prevent these complications, replacement therapy should be started as soon as possible (12). The key factor to successful treatment is educating the parents of hypothyroid children (14). The primary studies showed that early treatment before 3 months of birth is followed by normal values of IQ in 85% of infants, while in more than 80% of the infants with delayed treatment, IQ is reported to be lower than normal (14). Through neonatal screening program and timely diagnosis and proper treatment of sufferers, prognosis of infants with CH has improved dramatically (14). 

Before starting the neonatal screening program, the time of diagnosis was from one month to five years or more, so, the IQ of the patients with CH was dramatically reduced and IQ level was lower than 15 within 19 % of the patients (deep mental retardation). However, neonatal screening program with early diagnosis and treatment prevented irreversible and serious mental retardation (4). Several risk factors affecting the final outcome of psychological development in CH with early treatment have been investigated, including: the primary concentration of serum T4 before starting of treatment, the degree of neonatal skeleton development, etiology of CH, onset age of treatment, starting dose of levothyroxine, efficacy of replacement therapy within the first 2 years of life and since then, social and economic classes of family (15). 

Denver Developmental Screening Test II (DDST II) is one of the important and valid tests in evaluating and screening of growth and developmental matters from birth to six years old (16,17). The test includes 125 topics that fall into four groups: social– personal, fine motor, language, and gross motor like sitting, walking and jumping (18). With better and more accurate control of the disease, a normal growth and development is possible in majority of these patients, so that they can have a healthy life like other people.

Delayed treatment causes irreparable complications of the brain especially decreased level of IQ in patients. These brain consequences of hypothyroidism have many social, cultural and economic effects on health system, family and society. Since studies conducted in the world that have examined developmental condition in patients with CH, have had controversial results and besides, DDST II was not used very much to evaluate developmental status of infants suffering CH, we aimed to study the physical and developmental condition using DDST II (which is approved by the Ministry of Health and shows results in terms of quality and screening) (19), and analyze the degree of occurrence of mental retardation in these patients that the latter is the most important and main therapeutic goal in the treatment of patients with CH.

## Methods

**Study design and population: **In this case- control study with a sampling method of simple non- probability, 200 children aged 3-6 years were taken into consideration including two groups of case and control. The case group included 100 children referring to the Endocrine Clinic of Amirkola Children's Hospital Since 2011- 2017 that received levothyroxine pill with the diagnosis of CH. Control group included one hundred healthy children aged 3-6 years who were matched with the case group other than CH, referred to Endocrine Clinic of Amirkola Children's Hospital for growth monitoring. They had normal physical examinations and normal growth laboratory tests. 

**Methods: **The Denver developmental screening test-II (DDST_II) was used to assess the developmental factors in four areas of gross motor, fine motor, personal-social and language. Then, comparison was performed between two groups 

**Measuring tools: validity and reliability: **Data were collected using questionnaire, interview, observation and checklist. For both case and control groups, checklists have been filled out while referring to conduct DDST II. The study of developmental condition was conducted under the supervision of pediatric psychiatrist as well as filling out Denver test II questionnaire (in both case and control group) and by a pediatric resident. This test is applicable for children up to 6 years old and the time of each test is about 20 minutes (18). The test contains 125 items. Each item is scored as pass, fail, or refused. The cautions are considered when the items are completed by 75%-90% of patients but failed; If those are completed by 90% of children but failed are called delays. On the other hand, no delay in any domain and no more than one caution means a normal score; when, one or more delays or two or more cautions is seen, a suspect score is considered; Finally, an untestable score occurs during enough refused items that the score would be suspect if they had been delays (20).

**Intervention: **The children were studied in terms of nutrition types (breast milk or formula), height (centimeter) and weight (kg) and body mass index (BMI) (wt/[height]^2^). For definition the BMI status of children, the BMI standard curve of girls and boys 2-20 years old was used (< 5%: underweight, 5-85%: normal, 85-95%: overweight, > 95%: obese). Pediatric endocrinologist examined the clinical manifestation, physical examination and observation of thyroid function test. Pediatric neurologist studied neurologic status of the patients. 

At the time of patient referral, the initial level of TSH resulted from thyroid screening program as well as T4 and TSH levels related to venous blood sampling were recorded. For children with CH who were above 3 years of age, drug treatment was discontinued, therapy interruption took place for one month and TSH and T4 were measured again. Also, the TSH increased, T4 decreased, they were classified into permanent group and otherwise, they were considered as transient group. The case group had regular visits in an endocrine clinic. Control of disease and dose adjustment of levothyroxine were done accurately based on thyroid function test. Accordingly, all case groups were in the stable condition of thyroid function during this period.

**Ethical consideration: **Data were carefully collected and recorded. Gathered data were reviewed and possible errors were minimized. Iran health and training organization standard questionnaire was used. The importance of the subject was explained to the parents and it was accomplished through full satisfaction.

**Inclusion and exclusion criteria**

**Inclusion criteria:**

**Case group:** One hundred patients with diagnosis of CH between the ages of 3-6 years who referred to Endocrine Clinic of Amirkola Children's Hospital between 2011-2017 and were treated with levothyroxine tablets. 

**Control group:** One hundred healthy children aged 3-6 years

who were matched with the case group other than CH, referred to Endocrine Clinic of Amirkola Children's Hospital for growth monitoring. They had normal physical examinations and normal growth laboratory tests. 

**Exclusion criteria: **These cases were removed from the study: Genetic and metabolic illnesses and syndromes, severe unknown developmental delay, chronic or repetitive illnesses, suffering congenital and acquired diseases, prior hypoglycemia, taking medication like anticonvulsant drugs and corticosteroids, history of head trauma, history of neonatal asphyxia and electrolyte imbalances, history of neurological and psychiatric problems in the family, improper treatment of the CH including not taking medicine or taking it irregularly.

**Data Analyses: **Data were analyzed using SPSS 21 using descriptive statistics, t-test and chi- square. Z- Score was used to assess physical and mental conditions and based on data distribution, t-test was applied. Besides, chi-square was used to compare the results of screening test and thyroid venous blood tests and p<0.05 was considered statistically significant. 

**Laboratory measurements: **All infants suspected to CH in heel sample screening (TSH >5 MIU/L), were surveyed again by a Venus sampling and after confirmation of the disease (T4 <6.5µg/dl, TSH> 10 MIU/L) (21), treatment with levothyroxine pill was started immediately with the dose of 10-15 g/kg/day and according to the test conditions, the children were examined every 1 to 3 months and thyroid function test was reevaluated. 

## Results

In this study, a comparison was drawn between case and control group including 100 children aged 3 to 6 years for each group with average age of 54.62±15.72 and 59.68±15.64 months, respectively. Table 1 shows the frequency distribution and the percentage of demographic information of children in case and control groups. Demographic data included gender, nutrition, place of residence and pregnancy conditions. The number of premature birth was more than control group in case group with meaningful relationship. 

There were 47 girls and 53 boys in the case group and 52 girls and 48 boys in the control group. The mean and standard deviation of height, weight and BMI was 103.03±11.34 cm, 17.54±5.39 kg and 16.65±3.10 kg/m^2^ in the case group, respectively. However, in control group, it was 109.43±14.87 cm, 21.02±9.19 kg and 16.67±1.78 kg/m^2^, respectively. The average height was 50-75% in both groups in height standard curve; the average weight was 50-75% of weight standard curve in the case group and 75-90% in the control group (based on the age average). BMI was in normal range in both groups (5-85% of BMI standard curve). Table 2 shows frequency distribution and percentage of demographic information related to type of CH, thyroid screening test, and family background of thyroid in case group. 

Sixty- five percent of patients in the case group did not have associated symptoms before the diagnosis of CH. Related symptoms of 35 other patients are shown in table 2. Findings showed that the number of patients with permanent CH outnumbered its transient pair in case group (55% vs. 45%) and permanent CH in premature patients was higher than term patients .Also, 71 (71%) patients had positive neonatal screening test with negative screening test in 29 (29%) patients. The mean TSH level in transient and permanent group was 21.9±33.43 and 31.67±43.30 mIU/L, respectively with a meaningful relationship (P=0.003). The average of venous T4 level was 7.60±3.01 µg/dl in the case group with a lowest level of 1 and highest level of 16 µg/dl. Besides, the average serum level of TSH was 27.23±39.24 mIU/L (at least 2 and a maximum of 185 mIU/L). According to the consideration, gross motor was normal in both groups (case and control). The results of fine motor, social personal, and language have been shown in two groups in table 3. 

Six (3 term, 3 preterm)- 5 (4 term, 1 preterm) and 7 (5 term, 2 preterm) children in the case group had difficulty in terms of fine motor, social-personal and language, respectively. It should be noted that all disorders diagnosed by DSST II, were seen among boys (except gross motor that was normal in all cases). The onset age of treatment was 31.28±27.48 and 63.31±69.90 days in patients with positive and negative screening tests, respectively. The relationship between the onset age of treatment and Denver test results was illustrated in table 4. This table shows that beginning age of treatment in cases with impaired DSST II in patients with negative screening test was higher than patients with positive screening test, of course, this was not a meaningful issue statistically (p>0.05). Table 4 shows the relationship between the type of CH and developmental condition based on chi-square. As you see, the abnormal results of the Denver test were more frequent in permanent than transient CH. The final result of Denver test II was abnormal in 6 (6%) patients of case group.

**Table 1:** Frequency distribution and the demographic percentage of children in case and control group

**Demographic Data**		**Case group**		**Control group**		**P.value**
		**Absolute frequency**	**Frequency percentage**	**Absolute frequency**	**Frequency percentage**	
gender	Girl	47	47	52	52	0.47
	boy	53	53	48	48	
Pregnancy condition	Term	64	64	96	96	0.001
	Preterm	32	32	4	4	
	Multiple pregnancy	3	3	0	0	
	IVF	1	1	0	0	
Total		100	100	100	100	

**Table 2:** Frequency distribution and demographic data percentage related to children in case group

**Demographic** **Data**		**Case group**	
		**Absolute frequency**	**Frequency percentage**
The type of CH	Transient	45	45
	Permanent	55	55
First TSH level in screening test	Abnormal	71	71
	Normal	29	29
Family history of Thyroid disorders	Yes	38	38
	No	62	62
Without associated symptoms		65	65
With associated Symptoms	Prolonged jaundice	23	23
	Constipation	7	7
	Constipation with prolonged jaundice	2	2
	Skin dryness	2	2
	Growth retardation	1	1
Total		100	100

**Table 3:** Denver test results in case and control group

**Criteria**	**group**	**Normal**		**Abnormal**		P-Value
		**Absolute frequency**	**Frequency percentage**	**Absolute frequency**	**Frequency percentage**	
Fine motor	case	94	94	6	6	0.013
	control	100	100	-	-	
Personal-social	case	95	95	5	5	0.02
	control	100	100	-	-	
Language	case	93	93	7	7	0.007
	control	100	100	-	-	

* Gross motor was normal in all patients of study.

**Table 4:** Relation between the age of treatment initiation (day) and type of CH & Denver test result

**Denver test result** *		**Type of CH %** **(number)**		**screening(person) and age of initiation of treatment(day) ** **(median & SD)**	
		Transient	Positive	Positive	negative
Fine motor	normal	44 (97.8)	50(90.9)	68 (31.76±27.91)	26(66.23±73.29)
	abnormal	1(2.2)	5(9.1)	3(20.33±12.70)	3(38±13.85)
P-value		0.219		0.48	
Social_personal	normal	45(100)	50(90.9)	68(31.69±27.89)	27(65.78±71.91)
	abnormal	0(0)	5(9.1)	3(22±15.58)	2(30±0.00)
P-value		0.062		0.55	
Language	normal	43(95.6)	50(90.9)	68(31.69±27.89)	25(60.84±66.83)
	abnormal	2(4.4)	5(9.1)	3(22.00±15.58)	4(78.75±97.50)
P-value		0.453		0.55	

* Median age of treatment initiation in gross motor was 57.15±15.84

* Gross motor result was normal in all case.

## Discussion

 The major issue of this study was to examine the developmental condition of children with CH with Denver test II, that showed 94% of children with CH reported to be normal and 6% of them had abnormal test, meanwhile gross motor had normal pattern but abnormalities were seen in fine motor (6%), personal- social (5%) and language (7%).Few studies have examined the developmental conditions of children with CH. Unuvar et al.’s study determined the relationship between development and beginning dose, age, gender and the level of TSH using Denver test and showed 23% of the permanent CH and 10% of transient CH had impaired Denver test, while all of these issues were related to the delays above 2 months at the beginning of treatment (22). 

In this regard, Gulshan et al.'s study was conducted in 2011 with the aim of examining the neurological development of children aged 1-5 years with CH using Denver test. The patients were divided into two groups including early treated group (59 people) and delay treated group (21 people). They had interference in the early treated group in some items as gross motor, fine motor personal- social and language that had a population of 6, 8, 4 and 12 in each disorder, respectively. But, these items were 12, 15, 14 and 16 in the delay treated group, respectively. It showed that timely diagnosis and treatment had better effect on the results and development of the patients (23). Razavi et al.'s evaluated the developmental status of 78 children with an ASQ measurement score that 41% of whom had developmental disorders and concluded that early diagnosis and treatment with levothyroxine and its appropriate dose are the most important factors in the ASQ measurement score (2). The result of our study showed that nobody had difficulty in terms of gross motor but 6 persons (6%) in fine motor (who 5 patients with permanent CH), 5 persons in personal-social aspect (all of them, with permanent CH) and 7 persons in language (5 patients with permanent CH) had abnormal results, that these differences were meaningful. The results of other four studies showed brain developmental disorder in other aspects as changing the quality of life (scope of exhilaration, social function, emotional role and mental health) using Bayley and IQ test which is an indicative of more incidence of this disorder in patients with CH (24-27). Bulus study was conducted to examine the development of children with CH using Denver test that showed the most abnormalities in gross motor (28) of which the result was not consistent with our study and the most disorder was associated with the delay in the onset of treatment after 15 days that was unrelated to T4 level and starting dose of treatment. 

In a retrospective study, conducted on 37 children with CH with an average age of 3.2 months, a comparison was drawn between children with permanent CH and transient increased TSH group (hyperthyrotropinemia) in terms of time of diagnosis, gestational age, sex, symptoms, and developmental status. Developmental examination was conducted through Denver test and WISc- R. The most clinical manifestation in both groups was prolonged jaundice. There was not any difference in terms of Denver test and WISc-R between hyperthyrotropinemia and permanent CH (29). Of course, our study lasted shorter. On the other hand, the effects of proper treatment of CH on developmental status are shown better in a longer period of time. The beginning age of treatment was compared between positive and negative screening test groups in this study. On the average, the beginning age of treatment was higher in cases of negative screening test, of which the number of people with impaired Denver test was remarkable. Basically, their treatment was launched at an older age that it shows the need for accurate screening and timely treatment. This finding was similar to Unuvar and Gulshan studies in which the delay in timely diagnosis and treatment of CH was followed with abnormality in Denver test (22, 23). The impaired Denver test was predominant in boys in the present study, but this result was not seen in any other studies. So, the analysis of relationship between abnormality in Denver test and patient's gender in children with CH is recommended (22, 23, 30). In the current study, most people with impaired Denver test in CH group, had permanent CH that was similar to the results of the Unuvar et al.’s study(23.1% in permanent type ratio to 10% in transient type) (22). 

Yarahmadi et al.’s study was conducted in 2009 that the results were not consistent with this study. There was no meaningful difference in terms of IQ in health and CH children (4). The cause of this discrepancy, using IQ test for developmental assessment and also age range of 4-5 years in subjects, meanwhile, children with this age range in this study had normal Denver test. 

The common findings were without any signs at the time of diagnosis of CH but prolonged jaundice was the most prevalent finding. The clinical presentation was the same as Unuvar’s study whose jaundice was a prevalent phenomenon in both transient and permanent CH group. However, in Gulshan’s study, coarse face was a prevalent finding in both the early and delayed treatment's groups which does not go with our study (22, 25). Regarding weight and height growth in the current study, children with CH were at the normal limits compared to healthy children. But Faizi et al. conducted on 760 children with CH and 552 healthy children as a control group. There was abnormality in weight and height in children with CH compared to healthy kids that this difference faded away through an increase in age and good treatment (31). Heyerdahl’s study showed that linear growth reduced in CH children within the first 6 to 12 months but increased in next 12 months through treatment. It proved that thyroid hormone is necessary for growth within the first months of life (21). The number of people with permanent CH was higher than transient in our study. 

It was consistent with Unuvar’s study, which 76.3% of patients were permanent and 23.7% of them were transients, however Dalili et al.’s study in Gilan showed that 56.8% of them were transient and 43.2% were permanent (22, 30). The reasons for difference in rate of transient and permanent CH are the following factors: Alteration in overall prevalence of CH, difference in studies because of predisposing genetic factors, and the effect of environmental factors. TSH average of permanent group had a higher level compared to transient group. It was the same as Dalili's study, 88.03±38.22 and 21.27±14.25 mIU/L in permanent and transient group, respectively (30).

One of the strengths of our study was the use of the Denver test to assess the development of children with congenital hypothyroidism, which is less commonly used in the world to evaluate the developmental status of children, while this test was approved by the Ministry of Health and analyzed the degree of occurrence of mental retardation in these patients.

It is suggested that multicenter studies with large sample sizes be conducted. Also, it is recommended that different developmental assessment tests for comparison the results be performed, simultaneously.

**Limitations of the study: **Limitations of this study were non-cooperation of some patients for follow-up and incomplete information from previous tests for evaluation in some patients.

In conclusion, Neonatal screening of CH is one of the main achievements of medical science. This study showed that the abnormal Denver test was higher in permanent group compared to the transient group. It was also obvious that the beginning age of treatment in cases of abnormal Denver test was in an older age regarding normal screening compared to abnormal screening test. Thus, if the patients are not diagnosed, nor treated, or received late diagnosis and treatment, developmental process is disturbed, a result in delay in neural and physical growth.

The ultimate results from Denver test II for developmental assessment showed that 94% of children with CH possessed normal development. Hence, early diagnosis and timely treatment plays a key role in preventing this complication.
